# Emerging technologies of employing algae and microorganisms to promote the return-to-field of crop straws: A mini-review

**DOI:** 10.3389/fbioe.2023.1152778

**Published:** 2023-03-30

**Authors:** Qian Lu, Yu Xiao, Pengfei Wu

**Affiliations:** ^1^ School of Grain Science and Technology, Jiangsu University of Science and Technology, Zhenjiang, China; ^2^ College of Forestry, Nanjing Forestry University, Nanjing, China

**Keywords:** crop straw, algae, microorganisms, environment, agricultural resource

## Abstract

As an agricultural waste, crop straw enriched with a variety of nutrients is regarded as an important fertilizer resource. In the past, crop straw return-to-field played a key role in the sustainability of agricultural environment, but some problems, such as ammonia loss in ammoniation, low rate of straw decomposition, and high carbon footprint, attracted researchers’ attentions. In this paper, we propose three technical routes, including cyanobacteria-based ammonia assimilation, microorganisms-based crop straw pretreatment, and microalgae-based carbon capture, to address the aforementioned problems. Besides, challenges which may hinder the practical application of these technical routes as well as the potential solutions are discussed in detail. It is expected that this paper could provide new ideas to the practical application of crop straw return-to-field.

## 1 Introduction

As a large country with the highest population in the world, China puts a priority on crop production, which is accompanied by the yield of a huge amount of crop straws (Yan et al., 2019; Zhang et al., 2020; Wang et al., 2022). At present, the average annual straw production in China is higher than 1 billion tons, accounting for about one-third of the global straw production (He et al., 2022). In the past, crop straw, which was regarded as an agricultural waste, was often burned, resulting in serious atmospheric pollution and waste of resources (Singh et al., 2021). Recently, the return of crop straw to field as fertilizer is conceived and intensively studied as a promising technology to support the sustainable development of modern agriculture (Yan et al., 2019). Compared to the straw burning, return of crop straw to field has no detrimental effects on air quality and is able to improve soil fertility, maintain soil biodiversity, and promote plant growth (Ma et al., 2020a; Wang et al., 2021; Islam et al., 2022). For instance, after returning straw to fields, with the release of nutritional compositions in crop straw, soil organic carbon content increased significantly by 13.97% (Wang et al., 2020; Wang et al., 2021), while soil nitrogen (N) content and crop yield increased by 11% and 7%, respectively (Wang et al., 2015). Fast development of biotechnologies are promoting the wide application of crop straw return-to-field (Hans et al., 2019; Zhang B. et al., 2022). Owing to the positive effects of returning crop straw to fields on soil fertility, microbial diversity, and plant growth, Chinese government has made policies to support the return-to-field of crop straws (Yin et al., 2018).

Although a variety of methods and technologies were developed to promote the return-to-field of crop straw (Wang et al., 2013; Sun et al., 2014; Ma et al., 2020b), there are still some serious problems occurred in the return-to-field of crop straw (Supplementary Figure S1).

### 1.1 Waste of nitrogen resource in ammoniation process

As a process of treating crop straw with anhydrous ammonia, ammoniation, which has been widely applied before the return-to-field of crop straw, can improve the N content of straw and the nutritional values of straw-based fertilizer (Gordon and Chesson, 1983; Li et al., 2022). However, ammoniation of crop straw is accompanied with the emission of a huge amount of ammonia (Borhami et al., 1982; Gordon and Chesson, 1983). Gordon and Chesson (1983) reported that treatment of straw with anhydrous ammonia (40 kg t^−1^ straw dry matter) only increased the N content of straw by 13.9 g kg^−1^, suggesting that over 65% of ammonia was wasted in the ammoniation process. Hence, it is of importance to further improve the N conversion ratio of ammoniation process.

### 1.2 Low rate of straw decomposition

Optimization of tillage methods, in some cases, could not effectively accelerate the decomposition of crop straw (Yin et al., 2018). Wang et al. (2013) compared the effects of two tillage methods, including plowing tillage and no-tillage, on soil property in straw return-to-field experiment, discovering that the difference between these two methods in the improvement of soil total organic carbon (TOC) was not significant (Wang et al., 2013). Similar result was observed in the experiment which compared the effects of rotary tillage and plowing tillage on soil property (Zhu et al., 2014). Incomplete decomposition of crop straw can negatively impact the plant root penetration and the seedling growth (Su et al., 2016; Su et al., 2020).

### 1.3 Invasive emission of CO_2_

The increase of CO_2_ emission caused by the return-to-field of crop straw has not been fully addressed (Li H. et al., 2018; Ren et al., 2019; Ma et al., 2020a). Straw return-to-field inputs additional carbon sources to soil and increases microbial activity in the soil, resulting in significantly higher CO_2_ and methane emissions from the soil (Wang et al., 2018). It was reported that the rate of methane emission from rice fields increased by 210% due to straw return-to-field (Yan et al., 2005). In addition, Cui et al. (2017) pointed out that straw returning to the field has a significantly higher global warming potential (GWP) than conventional fertilization systems (Cui et al., 2017). Therefore, contribution of crop straw return-to-field to global warming has attracted researchers’ attentions (Hong et al., 2016; Liu et al., 2019; Ma et al., 2019).

Herein, we would like to focus on the recent progresses of employing algae and microorganisms to solve the aforementioned problems. In this work, a technical road-map for the sustainable development of straw return-to-field is presented and an in-depth discussion of the application of practically-feasible biotechnology is provided then. It is expected that this work can spur researchers to focus on the environmental problems occurred in the straw return-to-field and provide potential solutions to those problems for the development of eco-friendly agriculture.

## 2 Technical roadmap for the sustainable development of straw return-to-field

**FIGURE 1:**
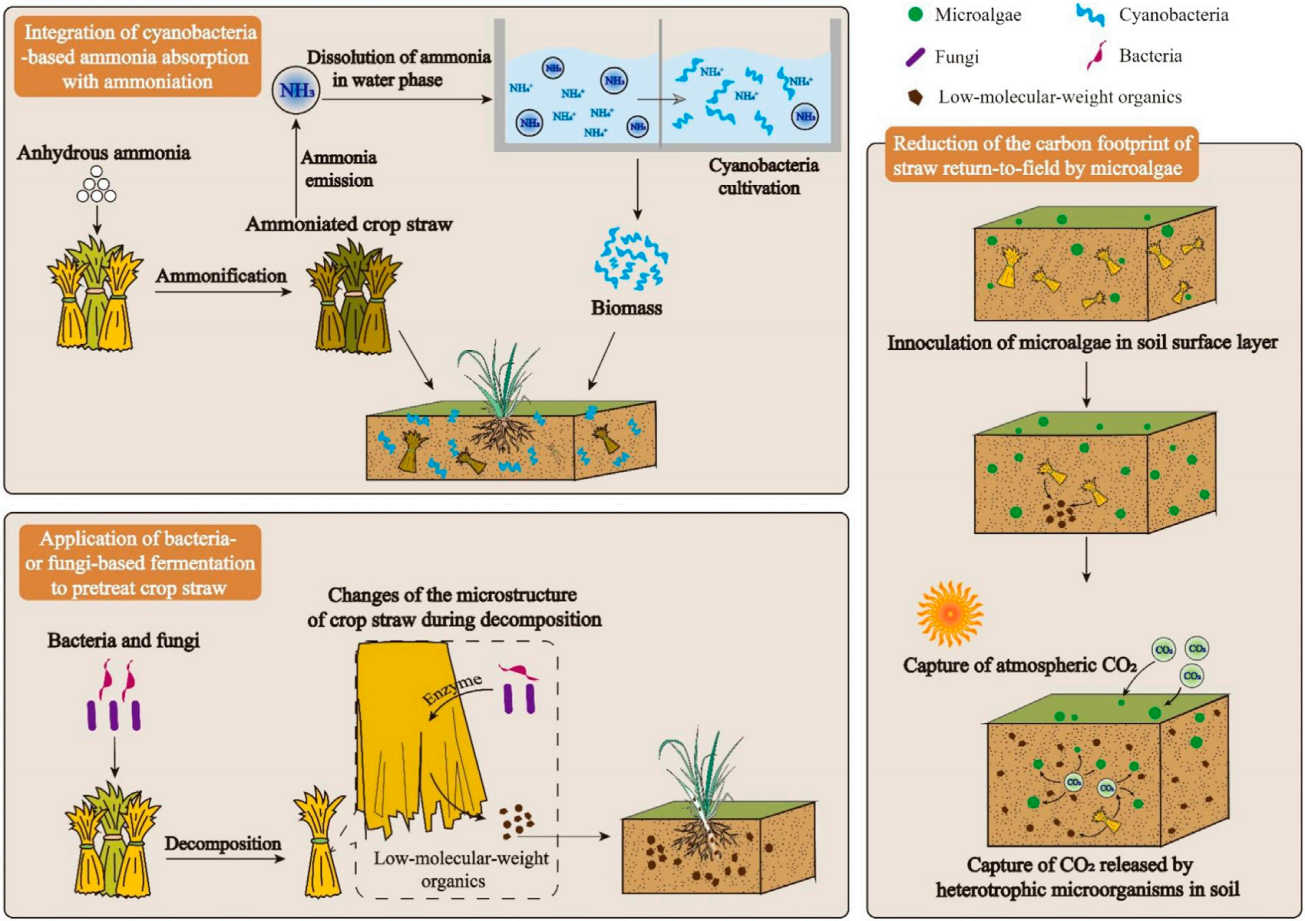
Technical roadmap for the sustainable development of straw return-to-field.

A road-map with three major technical routes is presented in Figure 1.

Firstly, to improve N conversion ratio of crop straw ammoniation, emitted NH_3_ can be collected and dissolved in water, which is further utilized for cyanobacteria cultivation. In cyanobacterial cells, assimilated ammonia is converted to algal protein (Lu et al., 2019). As the harvested algal biomass is added into soil as fertilizer, ammonia will be released back to soil with the decomposition of algal protein (Zou et al., 2021).

Secondly, microorganisms, particularly bacteria and fungi, are employed to pretreat crop straw. Extracellular enzymes secreted by those microorganisms can promote the decomposition of crops straw (Ji et al., 2018). With the assistance of enzymes-secreting microorganisms, the decomposition rate of crop straw can be improved. Accordingly, nutrients contained in crop straw can be utilized by plants through an efficient way.

Thirdly, microalgae are spread on soil as a potential strategy to attenuate the CO_2_ emission of return-to-field of crop straw. Microalgae on soil surface can capture CO_2_ *via* photosynthesis, creating a huge carbon sink (Lu et al., 2022; Lu and Xiao, 2022). With the photosynthesis of microalgae on soil surface, carbon footprint of crop straw return-to-field can be lowered.

The feasibility of aforementioned strategies is intensively studied. Firstly, cyanobacteria-based ammonia assimilation is applied in wastewater remediation to achieve the nitrogen recovery (Lu et al., 2017; Lu et al., 2019). Besides, cyanobacteria with high content of nitrogen have been proven to be a good resource for organic fertilizer (Acea, 2003; Abinandan et al., 2019). Secondly, mechanisms associated with the bacteria- or fungi-based crop straw decomposition have been intensively studied, demonstrating the possibility of using microorganisms to promote the crop straw return-to-field (Wood et al., 1986; Ji et al., 2018). Thirdly, microalgae have been intensively employed to reduce carbon emission of traditional factories. For example, some microalgal strains perform well in assimilating CO_2_ in flue gas, contributing to the construction of net-zero carbon factory (Cheah et al., 2015). Besides, mechanisms of algal photosynthesis and critical points enhancing the microalgae-based carbon fixation have been studied (Singh et al., 2016; Lu et al., 2018a; Petrova et al., 2020).

## 3 Integration of cyanobacteria-based ammonia absorption with ammoniation

### 3.1 Ammonia emission in ammoniation process

Ammoniation is conducted to generate ammonolysis reaction in crop straw by using ammonia-containing chemicals, such as liquid ammonia, urea, and ammonium bicarbonate (Yu et al., 2015; Doğan and Hayırlı, 2022). Through ammoniation, ester bonds between lignin and polysaccharide in crop straw can be damaged, making straw more decomposable in soil (Datsomor et al., 2022). Up to now, although key parameters, including temperature, moisture level, treatment period, and so on, have been optimized, ammonia emission in ammoniation process is still a serious challenge (Cloete and Kritzinger, 1984; Ma et al., 2020c). According to the study of Ma et al. (2020a) that used 5% urea (46% N content) for ammoniation, crude protein contents in rice straw (350 g) with ammoniation and without ammoniation were 9.27% and 5.79%, respectively (Ma et al., 2020c). If the nitrogen-to-protein conversion factor was set as 6.25, the addition of 5% urea (8.05 g N) in 350 g rice straw (3.24 g N) only increased the total N content of rice straw from 3.24 g to 5.19 g (Lu et al., 2015). In other words, 6.10 g N from urea was wasted in the ammoniation process while only 1.95 g N in urea was combined to rice straw (Ma et al., 2020c). Therefore, in this work, N conversion ratio of ammoniation was only 24.22% while about 75.78% of nitrogen in urea was wasted (Supplementary Figure S1).

Ammonia emission may dramatically increase the ammonia concentration in a closed environment, causing potential explosion under certain conditions (Li H. et al., 2018). Besides, ammonia released into atmosphere can be a promoter of the formation of haze (Ye et al., 2011). Hence, measures must be taken to attenuate the ammonia emission in ammoniation process. As shown in Figure 1, to reduce the ammonia emission in ammoniation process, a potential strategy of recovering the ammonia released from ammoniation process by growing cyanobacteria is proposed. In this strategy, ammonia released from ammoniation is collected to used as a nitrogen source of culture medium for cyanobacteria production. Then, cyanobacterial biomass enriched with nitrogen can be used as organic fertilizer to improve soil fertility.

### 3.2 Cyanobacteria-based ammonia absorption

Cyanobacteria are a group of blue-green algae with the great capacity of assimilating nitrogen for protein synthesis (Lu et al., 2019). In the metabolisms, nitrate in external environment must be converted to ammonia by nitrate reductase and nitrite reductase while ammonia can be directly assimilated by cyanobacterial cells (Li et al., 2016). Since ammonia in external environment can be efficiently used by cyanobacteria as a nitrogen source, cyanobacteria can be employed for ammonia removal. Previous studies have intensively cultured various species of cyanobacteria for ammonia recovery in different types of wastewater or culture media (Lu et al., 2019; Srimongkol et al., 2019). Cyanobacteria can survive in an ammonia-rich environment and perform well in ammonia removal (Supplementary Table S1). For example, in a synthetic wastewater containing about 206 mg/L ammonia, *Spirulina platensis* removed 80%–84% nitrogen. In water environment with low ammonia concentration, cyanobacteria could also efficiently fix ammonia (Wuang et al., 2016; Álvarez and Otero, 2020). Therefore, it is a practically-feasible method to use cyanobacteria for ammonia recovery.

Through glutamine synthetase-glutamine oxoglutarate aminotransferase (GS-GOGAT) pathway, absorbed ammonia can be converted to amino acids and protein by cyanobacteria (Lu et al., 2018a). Protein content in *Spirulina* sp., a typical species of cyanobacteria, could reach 59%–69% (Lu et al., 2019). If the nitrogen-to-protein conversion factor is set as 6.25, 1 kg cyanobacterial biomass (590–690 g protein) contains 94.4–110.4 g nitrogen, which is equal to 114.6–134.1 g ammonia. Accordingly, the cultivation of cyanobacteria can be regarded as an important process of the bio-conversion of ammonia to protein.

### 3.3 Utilization of cyanobacterial biomass as organic fertilizer

Protein-rich cyanobacterial biomass can be utilized as organic fertilizer, which can slowly release nutrients, including nitrogen, phosphorus, and organic carbon, to improve soil fertility (Malam Issa et al., 2006; Kumar et al., 2013). Compared to the direct use of ammonia solution as fertilizer, the use of cyanobacterial biomass as fertilizer has some advantages. Firstly, cyanobacteria-based fertilizer can release ammonia slowly with the gradual decomposition of cyanobacterial protein. Compared to chemical fertilizer, cyanobacteria-based fertilizer could release ammonia *via* a slow-release process (Lu and Xiao, 2022). The slow-releasing process of ammonia has not detrimental effects on crops but can continuously support the crop growth (Singh et al., 2019). Secondly, ammonia, which is slowly and continuously released from cyanobacterial biomass, can be assimilated by crops immediately. Under this situation, the loss of nitrogen resource caused by ammonia evaporation in fertilization process can be attenuated (Jhala et al., 2017; Lu et al., 2022).

As shown in Table 1, in the agricultural practice, with the addition of cyanobacteria in soil, soil fertility can be improved and the growth of plants can be enhanced (Abinandan et al., 2019). Firstly, due to the high content of protein in cyanobacteria, nitrogen content in soil can be improved after cyanobacteria inoculation (Acea, 2003). Besides, some cyanobacteria are able to fix atmospheric nitrogen during the fertilization process, further improving the nitrogen content in soil (Harding et al., 2018). The fixation of atmospheric nitrogen by cyanobacteria is another factor that contributes to the increase of nitrogen content in soil (Lu et al., 2022). Secondly, after cyanobacteria inoculation, microbial biomass carbon in soil can increase, contributing to the improvement of soil fertility (Karthikeyan et al., 2007; Song X. et al., 2021). Thirdly, cyanobacteria with filamentous cell structure and water retention ability can increase soil aggregate stability (Malam Issa et al., 2006). Previous studies have demonstrated the positive effects of cyanobacteria inoculation in soil on the growth of crops and vegetables (Karthikeyan et al., 2007; Kumar et al., 2013; Dash et al., 2016).

**TABLE 1:** Utilization of cyanobacteria as fertilizer to improve soil fertility and enhance plant growth.

Cyanobacteria	Fertilization information	Soil quality	Plant growth	References
*Anabaena azotica*	Replacement of chemical nitrogen fertilizer with nitrogen-fixing cyanobacteria by 30%, 50%, 70% and 100%	30% substitution of chemical nitrogen fertilizer with *Anabaena azotica* resulted in monetary savings of around 5.77 USD ha^-1^.	Substitution of chemical nitrogen fertilizer with *Anabaena azotica* increased rice gain yield, plant height, and the numbers of panicles and tillers.	Zhang et al. (2021)
*Nostoc carneum* and *Nostoc commune*	Addition of 12 g (wet weight) cyanobacterial biomass in 12 kg soil	—	The use of cyanobacteria as fertilizer improved the amount of spike per plant, amount of grains per spike, and total grain weight per spike.	Chittapun et al. (2017)
*Nostoc minutum* and *Anabaena spiroides*	Replacement of chemical fertilizers with 100% algae	(1) 100% substitution of chemical fertilizer with cyanobacteria increased nitrogen content in soil from 222.85 mg/kg to 305.10 mg/kg;	(1) 100% substitution of chemical fertilizer with cyanobacteria negatively impacted the vegetative growth of broad bean plant;	Al-Sherif et al. (2015)
		(2) 100% substitution of chemical fertilizer with cyanobacteria resulted in lower contents of phosphorus and potassium;	(2) The mixture of cyanobacteria with organic fertilizer had more positive effects on plant dry weight and shoot height than full chemical fertilizer and cyanobacteria.	
		(3) 100% substitution of chemical fertilizer with cyanobacteria dramatically reduced the contents of heavy metals, such as Pb, Cd, and Ni, in soil.		
*Anabaena azotica*	Addition of cyanobacteria: 150 kg/ha and 300 kg/ha	(1) Compared to the control with no nitrogen supply, addition of cyanobacteria increased the contents of total nitrogen, total phosphorus, and organic carbon in soil;	Compared to the control with no nitrogen supply, addition of cyanobacteria in soil improved rice yield to around 4,000 kg/ha.	Xuening Song et al. (2021)
		(2) Compared with the addition of urea, the fertilization with cyanobacteria reduced the average nitrogen (nitrate, ammonia, total dissolved nitrogen, and dissolved organic nitrogen) leaching loss.		
*Oscillatoria*, *Nostoc* and *Scytonema*	1×10^5^ and 2×10^5^ cyanobacteria g^-1^ soil	(1) Inoculation of cyanobacteria and mixed cyanobacteria increased nitrogen content in lime soil from 1.3 g/kg to 51.2–108.0 g/kg;	—	Acea (2003)
		(2) Inoculation of cyanobacteria in soil improved the contents of Ca^2+^, Mg^2+^, Na^+^, and K^+^.		
*Microcoleus vaginatus*, *Scytonema javanicum*, etc.	—	(1) Cyanobacterial inoculation treatment increased organic carbon content in soil from 0.33 to 0.49 g/kg to 2.67–2.71 g/kg;	—	Wang et al. (2009)
		(2) Cyanobacterial inoculation treatment increased total nitrogen content in soil from 0.18 to 0.19 g/kg to 0.43–0.45 g/kg		
		(3) Cyanobacterial inoculation treatment increased the ratio of C/N from 1.85 to 2.55 to 5.97–6.35.		
*Nostoc*	—	(1) Cyanobacteria inoculated on soil improved soil aggregate stability;	—	Malam Issa et al. (2006)
		(2) Cyanobacteria inoculated on soil improved water filtration.		
*Aphanothece* sp. and *Gloeotrichia* sp.	—	Cyanobacteria inoculation increased acetylene reduction activity, contributing to the improvement of nitrogenase activity in soil.	(1) Compared to no cyanobacteria inoculation, *Gloetrichia* inoculation and *Aphanothece* inoculation increased rice yield from 5.20 g/pot to 7.02 g/pot and from 5.20 g/pot to 5.49 g/pot, respectively;	Dash et al. (2016)
			(2) Compared to no cyanobacteria inoculation, *Gloetrichia* inoculation and *Aphanothece* inoculation increased the number of rice panicles from 5.66 pot^-1^ to 8.97 pot^-1^ and from 5.66 pot^-1^ to 6.99 pot^-1^, respectively.	
Calothrix ghosei, *Hapalosiphon intricatus* and Nostoc sp.	—	In glasshouse condition, cyanobacteria inoculation increased microbial biomass carbon in soil from 106.3 mg kg^-1^ soil to 159.0–227.0 mg kg^-1^ soil.	(1) In phytotron facility, cyanobacteria increased plant dry weight of wheat from 16.130 g pot^-1^ to 17.660–21.544 g pot^-1^.	Karthikeyan et al. (2007)
			(2) Cyanobacteria increased plant height and grain yield of wheat.	
*Anabaena laxa* and *Calothrix elenkinii*	1.6 μg chl g soil^-1^	—	(1) After cyanobacteria inoculation, plant fresh weight of coriander increased, reaching around 300 mg.	Kumar et al. (2013)
			(2) Cyanobacteria inoculation increased plant shoot length and plant root length.	
			(3) Cyanobacteria inoculation increased peroxidase activity in shoot and root.	

## 4 Application of microorganisms based fermentation to pretreat crop straw

### 4.1 Decomposition process of crop straw

Zhang et al. (2014) analyzed 784 crop straw samples, including cotton stalk samples, wheat straw, rape stalk, rice straw, and corn stover, collected from different regions of China, discovering that crop straw contains some essential elements, including carbon, nitrogen, sulfur, phosphorus, and potassium (Zhang et al., 2014; Wang et al., 2020). It was observed that in nature, crop straw can be decomposed gradually by bacteria, fungi, and other microbes with the assistance of hemicellulase, cellulase and ligninase (Henriksen and Breland, 2002; Jin et al., 2022). For example, Ji et al. (2018) reported that *Methanosarcina* and *Methanothrix* as well as hydrogentrophic *Methanocella* could be regarded as the important archaeal taxa involved in rice straw degradation (Ji et al., 2018).

Decomposition of crop straw by microorganisms-based fermentation can be divided into two major steps (Guo et al., 2018; Gao et al., 2016). Firstly, microorganisms secret extracellular enzymes, particularly cellulase and ligninase, which can break the chemical structure of crop straw, converting high-molecular-weight organics to low-molecular-weight organics (Xiong et al., 2014; Harindintwali et al., 2020). Secondly, after the enzymes-driven crop straw decomposition, nutrients of crop straw could be assimilated by microorganisms (Zhao et al., 2019). In this step, some microbial strains may secret organic acids with low-molecular-weight. Both two steps could play a key role in the improvement of soil fertility. On one hand, extracellular enzymes secreted by microorganisms could promote the decomposition of crop straw and accelerate crop straw return-to-field. On the other hand, organic acids secreted by some microbial strains could increase the organic carbon content in soil (Zhang Y. et al., 2022).

### 4.2 Microorganisms-based fermentation for crop straw pretreatment

Baige Zhang et al. (2022) cultivated *Trichoderma reesei* to pretreat corn straw before return-to-field, discovering that the humic acid carbon content in fungi-treated corn straw was much higher than that in untreated corn straw (Zhang Y. et al., 2022). Fermented corn straw treated with *T. reesei* is more conducive to increasing the content of easily oxidizable organic carbon in soil than direct application of corn straw. In addition, some conditions, such as temperature, O_2_ concentration, and air humidity, which can determine the fermentation process, could be optimized to enhance the performance of crop straw pretreatment (Yu et al., 2019; Mengqi et al., 2021). Last but not the least, some studies focused on the emission of greenhouse gases (GHG) during the decomposition of crop straw in soil (Devêvre and Horwáth, 2000). In the real-world application, it is also important to take measures to reduce the GHG emission during the microorganisms-based crop straw pretreatment.

At present, although the research directly related to bacteria- or fungi-based crop straw pretreatment is very limited, there are some studies focusing on microorganisms screening and major components of crop straw. Firstly, in the past decades, various microbial strains, including *Bacillus subtilis*, *Neocallimastix frontalis*, *N. frontalis*, *Pleurotus* sp., *T. reesei*, and so on, with the ability of secreting extracellular cellulase and ligninase were discovered and isolated (Wood et al., 1986; Singh et al., 2013; de Paula et al., 2019; Rathnan and John, 2021). In the practical application, to enhance the performance of crop straw pretreatment, a mixture of microbial strains with different extracellular enzymes could be applied. Secondly, major components of crop straw have been intensively studied, providing guidelines for the screening of microorganisms (Tamaki and Mazza, 2010). For example, to promote the decomposition of crop straw with high contents of cellulose, microbial strains secreting extracellular cellulase should be selected for the pretreatment.

## 5 Reduction of the carbon footprint of straw return-to-field by microalgae

### 5.1 Carbon footprint of straw return-to-field

Due to the high content of lignin, cellulose, and hemicellulose in crop straw, the decomposition of straw in soil could release a large amount of carbon (Supplementary Table S2). Gan et al. (2011) reported that straw and root decomposition could emit 120 kg CO_2_-eq ha^−1^, suggesting that straw in soil can be a source of carbon emission (Gan et al., 2011). Wu et al. (2022) reported that carbon footprint of wheat cultivation supplied with straw and chemical fertilizer reached 1978.72 kg CO_2_-eq ha^−1^ and carbon efficiency was only 11.87% (Wu et al., 2022). In addition to carbon footprint of single crop production, carbon footprint of crops rotation could be improved by the utilization of straw-based fertilizer. Shi-hao Li et al. (2020) found that in rice-wheat rotation, annual CH_4_ emission increased by 5.4%–72.2% with the straw return-to-field and carbon emission (4,275–4,989 kg CO_2_-eq ha^−1^) also increased with increasing straw returning amounts (Li S. H. et al., 2020). Similar result confirming the positive correlation between straw and soil carbon footprint was also discovered by Xuening Song et al. (2021) that assessed the effects of straw retention and straw removal on carbon footprint of continuous corn cropping and corn-soybean rotation (Song Q. et al., 2021). Hence, straw return-to-field usually gives rise to GHG emissions from the soil and increase carbon footprint of agricultural activity (Dhaliwal et al., 2019).

### 5.2 Microalgae-based carbon assimilation in soil

Microalgae, which can efficiently assimilate atmospheric CO_2_ and organic carbon, are widely spread in the river, desert, lake, and ocean. It was reported that carbon content in *Chlorella pyrenoidosa* fell in a range of 47.00%–56.02% (Huang et al., 2015). If CO_2_ is the sole carbon source of microalgae, the accumulation of 1 kg microalgae biomass means that 1.477–1.761 kg CO_2_ has been assimilated. According to the study of Lu et al. (2018a), the maximum O_2_ productivity of 8 g microalgae on biofilm (200 cm^2^) could reach 249.44 mg h^−1^ (Lu et al., 2018b). In other words, 1 kg microalgae could assimilate CO_2_ at a maximum rate of 42.87 g h^−1^. Hence, microalgae culture can be regarded as an efficient way for carbon capture. Since microalgae perform well in carbon fixation and can be easily obtained, it may be a practically-feasible way to use microalgae to reduce carbon footprint in agricultural activity.

The reduction of carbon footprint of soil by living microalgae might be attributed to two mechanisms (Figure 1). Firstly, living microalgae on the surface of soil can capture atmospheric CO_2_ through photosynthesis. If the environmental factors, such as temperature, sunlight, and moisture, are suitable to the algal photosynthesis, microalgae on the surface of soil can be regarded as a carbon sink, which continuously convert atmospheric CO_2_ to biomass (Abinandan et al., 2019). In this case, microalgae could reduce the total carbon emission of soil by creating a carbon-negative micro-environment on the surface of soil. Secondly, microalgae could establish synergistic relations with soil bacteria and fungi for carbon utilization. Soil microorganisms, including microalgae, bacteria, and fungi, can form consortia, in which microbial cells not only interact physically, but also exchange nutrients (Perera et al., 2018). For example, CO_2_ released by soil bacteria and fungi can be immediately captured by microalgae in the consortia. In this way, carbon emission from the heterotrophic metabolisms of soil bacteria and fungi can be reduced to some extent.

In the practical application, some important issues merit the researchers’ attentions. Firstly, although the effects of alien microalgal species on soil environment have not been fully studied yet, potential threats of biological invasion caused by the use of alien microalgal species should be avoided (Korneva, 2014). Hence, native microalgal species, instead of alien microalgal species, should be isolated and cultured for carbon footprint reduction. Secondly, to improve the survival ratio of microalgae added in soil, soil properties, such as salinity, temperature fluctuation, moisture and so on, must be taken into consideration. For example, to reduce the carbon footprint of saline-alkali land, microalgal species with high tolerance to salt should be screened and then inoculated in soil (Qiao et al., 2015). Thirdly, to support microalgae growth in soil and promote carbon fixation, microalgae growth-promoting bacteria can be co-inoculated with microalgae in soil. It was discovered that some bacteria could promote the growth of microalgae by producing and secreting indole acetic acid (Dao et al., 2018). With the faster reproduction of microalgae in soil, a larger carbon sink can be created for carbon reduction.

## 6 Challenges and prospects

In practice, some challenges to these strategies are discussed and potential solutions are provided as well.

Firstly, it is not easy to improve ammonia assimilation ratio during cyanobacteria cultivation. Theoretically, cyanobacteria could efficiently assimilate dissolved ammonia in media. However, cyanobacteria growth could continuously increase the pH of media, disturbing the equilibrium of H^+^ and OH^−^. The pH of media with *Spirulina* cultivation could reach over 11, creating an alkaline environment (Pagels et al., 2019; Li H. et al., 2020). As a result, a high portion of ionized ammonium will be converted to unionized ammonia, causing ammonia emission. In the view of the present authors, to improve the ammonia assimilation ratio during cyanobacteria growth, pH of culture media should be strictly controlled to prevent the continuous increase of pH. For example, buffer can be added in culture media to attenuate the conversion of ionized ammonium to unionized ammonia during cyanobacteria growth. In addition, crop straw ammoniation and cyanobacteria cultivation can be performed simultaneously to ensure that ammonia released from ammoniation process can be immediately assimilated by cyanobacteria.

Secondly, in agricultural practice, due to the harsh environment, such as temperature fluctuation between day and night, microorganisms-based fermentation may be hindered. In addition, if microbial strains could not be preserved properly, their bio-activity may be negatively impacted. Therefore, measures must be taken to enhance the microorganisms-based crop straw pretreatment. Firstly, microbial strains should be preserved properly to prevent the decrease of microbial bio-activity and the degradation of microorganisms. Secondly, the optimal environment should be identified according to the biological characteristics of microorganisms. Then, the pretreatment of crop straw should be conducted under the optimal conditions to ensure that a higher ratio of nutrients in crop straw can be converted to low-molecular-weight nutrients. Thirdly, physical pretreatment or chemical pretreatment can be integrated with microorganisms-based crop straw pretreatment. For example, physical crushing could increase the total surface area of crop straw and enhance the interaction between microorganisms and crop straw.

Thirdly, production cost of microalgae must be further reduced to lower the total cost of the reduction of carbon footprint of crop straw return-to-field. Due to the high cost of culture media, artificial illumination, and biomass harvesting, the utilization of microalgae is seriously hindered in many sectors of agriculture and industry. Acien et al. (2012) reported that the unit production cost of microalgae biomass (dry weight) could reach 96 €/kg and the simplification of technology could reduce the unit production cost of microalgae biomass to 12.6 €/kg (Acien et al., 2012). In our view, some measures can be taken to lower the production cost of microalgae. Firstly, wastewater enriched with nutrients can be employed to cultivate microalgae (Xu et al., 2020; Zhong et al., 2021). Compared to artificial culture media, wastewater can be obtained at much lower cost, reducing the production cost of microalgae. Secondly, advanced technologies or equipment can be adopted to simplify the microalgae harvesting process. For example, compared to centrifugation, immobilized microalgae attached on biofilm can be harvested by using scrappers with much lower energy consumption and cost (Hu et al., 2021). Thirdly, photovoltaic technology can be employed to reduce the energy consumption caused by illumination. With the adoption of photovoltaic technology, solar energy can be stored in the form of electrical energy to provide illumination for microalgae growth at night (Zahedi, 2011). Thus, the energy consumption and cost of microalgae production can be reduced.
