# Supplementary material for: Emerging technologies of employing algae and microorganisms to promote the return-to-field of crop straws: A mini-review
Source: Front Bioeng Biotechnol. 2023 Mar 30;11:1152778. doi: 10.3389/fbioe.2023.1152778 (PMC10097884; doi:10.3389/fbioe.2023.1152778)
Supplement: Supplementary file 1 [file DataSheet1.doc]

**Table S1. Cyanobacteria-based ammonia assimilation**

| **Species of cyanobacteria** | **Culture medium** | **Cultivation time (day)** | **Initial concentration of ammonia (mg/L)** | **Removal efficiency of ammonia** | **Reference** |
| --- | --- | --- | --- | --- | --- |
| *Spirulina* sp. | Artificial effluent | 12 | 127.5 | ~ 100% | [1] |
| *Spirulina* sp. | Diluted brewery effluent | 5 | ~ 28 | 89.70% | [2] |
| *Spirulina platensis* | Synthetic wastewater | 15-25 | ~ 206 | 80-84% nitrogen removal | [3] |
| *Nostoc muscorum* & *Anabaena subcylindrica* | Sewage and industrial wastewater | 10 | 12.4 | 79.0% | [4] |
| *Nostoc* sp. | Industrial wastewater | 2 | ~ 12 | 84.1% | [5] |
| *Spirulina platensis* | Aquaculture wastewater | 13 | / | 87.77% | [6] |
| *Spirulina subsalsa* | Food industry effluent | 6 | 62 | 98.8% | [7] |
| *Spirulina subsalsa* | Food industry effluent | 6 | 31 | 99.9% | [7] |
| *Spirulina platensis* | Synthetic human urine | 7 | 50 | 97% | [8] |
| *Spirulina* sp. | Aquaculture wastewater | 3 | 2.56 | ~ 100% | [9] |
| *Nostoc* sp. | Municipal wastewater | 10 | ~ 24 | 84.12-95.17% | [10] |
| *Nostoc muscorum* | Kitchen waste water | 15 | 9.00 | ~ 93.3% | [11] |
| *Anabaena variabilis* | Food and dish washing wastewater | 15 | 7.3 | ~ 86.3% | [12] |
| *Nostoc muscorum* | Food and dish washing wastewater | 15 | 7.3 | ~ 98.4% | [12] |

**Table S2. Carbon footprint of farmland supplied with crop straw as fertilizers in China**

| **Location** | **Agricultural activity** | **Supplementation of crop straw** | **Annual carbon footprint** | **Reference** |
| --- | --- | --- | --- | --- |
| Anhui, China | Wheat cultivation | Wheat straw was supplied at 7500 kg ha^-1^ year^-1^ | 1978.72 kg CO_2_-eq ha^-1^ | [13] |
| Northeast China | Continuous corn cropping | Straw retention | 2707 kg CO_2_-eq ha^-1^ | [14] |
| Northeast China | Corn-soybean rotation | Straw retention | 1721 kg CO_2_-eq ha^-1^ | [14] |
| Hubei, China | Two-year cycle rice-wheat rotation | Straw was supplied at 6.56-7.41 t ha^-1^ year^-1^ and 4.13-5.04 t ha^-1^ year^-1^ in the rice- and wheat-growing seasons, respectively | 1.31 kg CO_2_-eq kg^-1^ grain | [15] |
| Shananxi, China | Wheat-maize double cropping | Maize straw was crushed into about 10 cm pieces and incorporated into soil (20 cm depth) and wheat straw was distributed on soil surface uniformly | Net carbon footprint: ~8 Mg CO_2_-eq ha^-1^ | [16] |
| Shananxi, China | Wheat-maize double cropping | Single straw return of maize | Net carbon footprint: ~9 Mg CO_2_-eq ha^-1^ | [16] |
| Shananxi, China | Maize cultivation | Ditch-buried straw returning with ridge-furrow plastic film mulch | ~403.03 kg CO_2_-eq ha^-1^  (Total greenhouse gas emissions: 4510.77 kg CO_2_-eq ha^-1^) | [17] |
| Gansu, China | Wheat-maize intercropping in arid areas | No-tillage with straw mulching and no-tillage with straw standing | 10495-10779 kg CO_2_-eq ha^-1^ | [18] |
| Hubei, China | Integrated rice-crayfish system | Crayfish feeding with straw returning | 18797 kg CO_2_-eq ha^-1^ | [19] |

**Figure caption**

Figure S1. Major problems of in the process of crop straw ammoniation


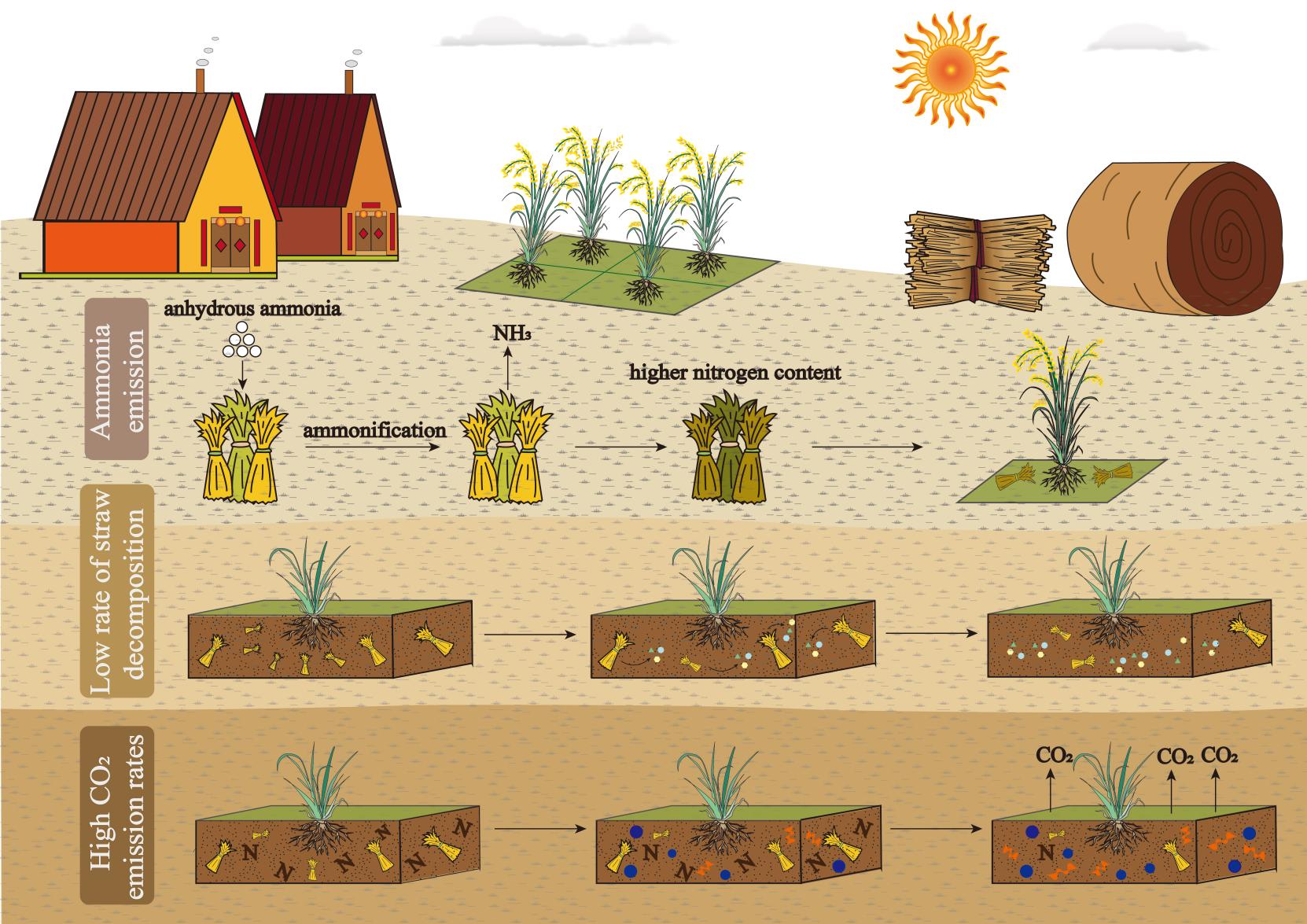


**Figure S1**

**Reference:**

[1] Q. Lu, P. Han, F. Chen, T. Liu, J. Li, L. Leng, J. Li, W. Zhou, A novel approach of using zeolite for ammonium toxicity mitigation and value-added Spirulina cultivation in wastewater, Bioresour Technol, 280 (2019) 127-135.

[2] Q. Lu, H. Liu, W. Liu, Y. Zhong, C. Ming, W. Qian, Q. Wang, J. Liu, Pretreatment of brewery effluent to cultivate Spirulina sp. for nutrients removal and biomass production, Water Sci Technol, 76 (2017) 1852-1866.

[3] X. Yuan, A. Kumar, A.K. Sahu, S.J. Ergas, Impact of ammonia concentration on Spirulina platensis growth in an airlift photobioreactor, Bioresour Technol, 102 (2011) 3234-3239.

[4] M.M. El-Sheekh, W.A. El-Shouny, M.E. Osman, E.W. El-Gammal, Treatment of sewage and industrial wastewater effluents by the cyanobacteria Nostoc muscorum and Anabaena subcylinderica, Journal of Water Chemistry and Technology, 36 (2014) 190-197.

[5] X. Álvarez, A. Otero, Nutrient removal from the centrate of anaerobic digestion of high ammonium industrial wastewater by a semi-continuous culture of Arthrospira sp. and Nostoc sp. PCC 7413, Journal of Applied Phycology, 32 (2020) 2785-2794.

[6] M. Wijayanti, D. Jubaedah, N. Gofar, D. Anjastari, Optimization of Spirulina platensis Culture Media as an Effort for Utilization of Pangasius Farming Waste Water, Sriwijaya Journal of Environment, 3 (2018) 108-112.

[7] L. Jiang, H. Pei, W. Hu, Y. Ji, L. Han, G. Ma, The feasibility of using complex wastewater from a monosodium glutamate factory to cultivate Spirulina subsalsa and accumulate biochemical composition, Bioresour Technol, 180 (2015) 304-310.

[8] Y. Chang, Z. Wu, L. Bian, D. Feng, D.Y.C. Leung, Cultivation of Spirulina platensis for biomass production and nutrient removal from synthetic human urine, Applied Energy, 102 (2013) 427-431.

[9] S.C. Wuang, M.C. Khin, P.Q.D. Chua, Y.D. Luo, Use of Spirulina biomass produced from treatment of aquaculture wastewater as agricultural fertilizers, Algal Research, 15 (2016) 59-64.

[10] S. Silambarasan, P. Logeswari, R. Sivaramakrishnan, B. Kamaraj, N.T. Lan Chi, P. Cornejo, Cultivation of Nostoc sp. LS04 in municipal wastewater for biodiesel production and their deoiled biomass cellular extracts as biostimulants for Lactuca sativa growth improvement, Chemosphere, 280 (2021) 130644.

[11] T. Vijaya, K.C. Mouli, S.D.S. Murthy, Biomass production and treatment of kitchen waste water with Nostoc muscorum ― a potential biofertilizer, Journal of Environmental Science for Sustainable Society, 5 (2011) 22-26.

[12] V. Tartte, C.M. Kalla, D.S.M. Sistla, Comparative studies on growth and remediation of waste water by two cyanobacterial biofertilizers, Agriculturae Conspectus Scientificus, 75 (2010) 99-103.

[13] L. Wu, X. Zhang, H. Chen, D. Wang, M.M. Nawaz, F. Danso, J. Chen, A. Deng, Z. Song, H. Jamali, C. Zheng, W. Zhang, Nitrogen Fertilization and Straw Management Economically Improve Wheat Yield and Energy Use Efficiency, Reduce Carbon Footprint, Agronomy, 12 (2022).

[14] Q. Song, J. Zhu, Z. Gong, Y. Feng, Q. Wang, Y. Sun, X. Zeng, Y. Lai, Effect of straw retention on carbon footprint under different cropping sequences in Northeast China, Environ Sci Pollut Res Int, 28 (2021) 54792-54801.

[15] S.-h. Li, L.-j. Guo, C.-g. Cao, C.-f. Li, Effects of straw returning levels on carbon footprint and net ecosystem economic benefits from rice-wheat rotation in central China, Environmental Science and Pollution Research, 28 (2020) 5742-5754.

[16] S. Li, M. Hu, J. Shi, X. Tian, J. Wu, Integrated wheat-maize straw and tillage management strategies influence economic profit and carbon footprint in the Guanzhong Plain of China, Sci Total Environ, 767 (2021) 145347.

[17] M. Zhang, X. Han, P. Dang, H. Wang, Y. Chen, X. Qin, K.H.M. Siddique, Decreased carbon footprint and increased grain yield under ridge-furrow plastic film mulch with ditch-buried straw returning: A sustainable option for spring maize production in China, Sci Total Environ, 838 (2022) 156412.

[18] W. Yin, Q. Chai, Z. Fan, F. Hu, H. Fan, Y. Guo, C. Zhao, A. Yu, Energy budgeting, carbon budgeting, and carbon footprints of straw and plastic film management for environmentally clean of wheat-maize intercropping system in northwestern China, Sci Total Environ, 826 (2022) 154220.

[19] L. Lin, S. Yanju, X. Ying, Z. Zhisheng, W. Bin, L. You, S. Zichuan, Z. Haoran, Z. Ming, L. Chengfang, W. Jinping, J. Yang, A. Maimaitizunong, C. Cougui, Comparing rice production systems in China: Economic output and carbon footprint, Sci Total Environ, 791 (2021) 147890.
